# Intracranial calcifications - the eternal companions

**DOI:** 10.1590/0100-3984.2020.53.4e2

**Published:** 2020

**Authors:** Fabio Noro

**Affiliations:** 1 Universidade Federal do Rio de Janeiro (UFRJ), Rio de Janeiro, RJ, Brazil. Email: fncursos@gmail.com.

Our great masters of neuroradiology, who lived in an era before the advent of computed tomography (CT), like to say that they are from an era when the skull was “hollow” for the radiologist. They also report that the only structures that allowed them to have a rough idea of what was happening inside the intracranial vault were calcifications. The deformations that the diseases caused in the brain were observed directly or indirectly on cerebral arteriography and pneumoencephalography. However, noninvasive analysis of the intracranial vault was productive only when there were intracranial calcifications to help them understand what was happening. At that time, the essential aspects in the interpretation of X-rays of the skull were not only pathological calcifications but also physiological ones, because a change in the position of a known calcified structure could represent deformation caused by a mass effect. On frontal and lateral X-rays of the skull, the neuroradiologists of the past looked for deviation of the calcified pineal gland to diagnose an expansile lesion of any kind-tumors or (in cases of blunt trauma) parenchymal/extra-axial hematomas. Pineal deviation as a sign of an expansile lesion was first described in 1912 by Schüller^(1)^, in a patient with a primary tumor. It was long used as one of the main tools of the trailblazing neuroradiologists.

In addition to the pineal gland, several other anatomical structures are physiologically calcified, such as the habenulae, meninges, and choroid plexuses. In some of those, the calcification process begins as early as childhood. Positional deviations of these other calcifications were also used as references in the diagnosis of expansile lesions, as was done with the calcified pineal gland. In 1941, Childe^(2)^ described the process of diagnosing expansile lesions in eight patients, using deviated choroid plexuses as a reference.

Direct diagnoses of certain diseases could also be made when the lesions had calcifications. Clustered calcifications, located at the periphery of the brain, were suggestive of meningiomas or osteomas. Calcifications in the middle of the brain suggested tumors, such as oligodendrogliomas. In the suprasellar region, calcifications could indicate a craniopharyngioma. In children, calcifications in the posterior fossa were diagnostic of medulloblastoma. The suspicion of an aneurysm was raised when nodular structures with peripheral calcifications were observed along the course of arteries.

For many years, intracranial calcifications were the main companions of neuroradiologists. CT and magnetic resonance imaging (MRI) gave neuroradiologists the advantage that had previously belonged exclusively to neurosurgeons: a view of the entire brain. For visualizing calcifications, especially tiny calcifications, CT is better than is MRI. That disparity was lessened by the advent of gradient-echo sequences, which improved the performance of MRI^(3,4)^.

The standard classification of intracranial calcifications is intra-axial or extra-axial. Among physiological calcifications, extra-axial calcifications are much more common, because of the location of the structures that usually calcify: the pineal gland, choroidal plexuses, and meninges. However, when these calcifications are more pronounced than usual, they suggest disease, as in cases of calcified choroid plexus papillomas^(5)^. Among the extra-axial lesions that frequently calcify, meningiomas merit special attention. More than 60% of meningiomas exhibit some calcification, whether granular, diffuse, peripheral, or complete. Besides, the presence of these tumors near the bone induces another type of pathological calcification, which is focal hyperostosis^(6)^. The most common physiological intra-axial calcifications are those of globus pallidus, habenulae, and cerebellum. However, when very pronounced, they are suggestive of pathologies such as Fahr’s disease^(6,7)^.

Many intra-axial lesions, including neoplastic, vascular, infectious, congenital, and metabolic lesions, can be calcified. In some cases, the presence of calcification changes the line of reasoning for the differential diagnosis. For example, if a primary frontal neoplasia has calcification, oligodendroglioma should be suspected. In addition, calcified metastases are suggestive of an osteogenic or mucinous tumor^(4,6)^.

The revolution caused by CT and MRI relegated calcifications to a supporting role. However, as they came to be better perceived, studied, and described, they were able to reclaim the role of the protagonist in some clinical situations. It is precisely in this context that the pictorial essay by Guedes et al.^(8)^, published in this issue of **Radiologia Brasileira**, makes a valuable contribution. With a particular emphasis on the characteristics of calcifications, the authors describe important details that facilitate the differential diagnosis.

Despite the tremendous technological advances in the imaging of the central nervous system, intracranial calcifications are still eternal companions in the daily lives of neuroradiologists.
